# Supplementary material for: Machine learning analysis of the UK Biobank reveals IGF-1 and inflammatory biomarkers predict Parkinson’s disease risk
Source: PLoS One. 2023 May 9;18(5):e0285416. doi: 10.1371/journal.pone.0285416 (PMC10168570; doi:10.1371/journal.pone.0285416)
Supplement: Attachment — Submitted filename: PLOS ONE reviewer responses_MA.docx [file pone.0285416.s002.docx]

Dear Prof. Sabbatinelli,

We re-submit our revised article entitled **“Machine Learning Analysis of the UK Biobank Reveals IGF-1 and Inflammatory Biomarkers Predict Parkinson’s Disease Risk”** for consideration for publication in PLOS ONE. We believe our article will be of great interest to your readers since it applies cutting edge machine learning to identify novel risk factors in Parkinson’s Disease (PD).

We would like to take this opportunity to thank the reviewer for their insightful feedback. We have responded to their comments point by point below. We believe these revisions have led to a significant improvement in the manuscript.

We would also like to thank you for the opportunity to revise and re-submit our manuscript and look forward to hearing your evaluation.

Yours sincerely,

Michael Allwright

**Response to reviewer**

#1: 1. The authors should have included the details of several analysis methods in the methodology part of the manuscript, which are essential for the analysis evaluation.

1.1 **Please provides hyperparameter tuning methods.**

*Additional details of the hyperparameter tuning method used have been added to the revised manuscript.*

1.2 **Please specify the AUC means comparison methods in figure 3.**

*Details of the statistical comparison of the AUC means between each model have been added to the methods. The statistical test used, and the result of the AUC means comparison has also been added to the figure legend for Figure 3.*

1.3 **Please specify the statistical methods used in figures 5, 6, 7, 8, and 9.**

*This information is contained in the methods:*

*“For both the male and female cohorts unpaired 2-sample t-tests were performed to compare the means for each variable in the non-PD group with the means of each variable at each disease stage: 5-10 years prior to disease diagnosis, 0-5 years prior to disease diagnosis and 0-5 years post disease diagnosis respectively.”*

*Also the statistical test used and the significance levels have been added to each figure legend.*

*In addition, the reporting of the statistics in the results have been simplified to report, P<0.05, P<0.01 or P<0.001 rather than specific p values, and some errors have been corrected, e.g. greater than sign was incorrectly used instead of less than sign.*

2. **After imputation, data duplication-checking might be needed.**

*Thank you for raising this concern. Data duplication checking was performed as one of the checks to confirm the pipeline was working as planned. We confirm that the datasets used post imputation contained no duplicate values. We had already pre-selected variables which were populated for >80% of participants. Also, our section on stratification by gender and disease progression (Figures 5,6,8 and 9) used non-imputed data and excluded null values from the analysis.*

3. **Please provide the definition of "overall health rating". Is this feature independent from other features?**

*This is a UKB questionnaire field (Data-Field 2178) and is self-reported by the participant on a 4-point scale from “poor” to “excellent”. It is independent of other features.*

4**. What is the meaning of "the unbiased IDEARS model"? What is the difference from the IDEARS model? How to define the unbiased property.**

*There is no difference between the two, and we apologise for this error. We have revised the wording in the manuscript to reflect this. We have also spelt out IDEARs as The Integrated Disease Explanation and Risk Scoring platform, an omission in the previous manuscript, so we hope all is clear now.*

5. **Is it possible to provide the list of features; Va, and Vc?**

*The list of features has been provided as a supplementary file supp_file1.xlsx.*

6**. Is it possible to provide the code of analysis pipeline?**

*The location code of the analysis pipeline is provided in the methods, and can be found at this link:* *https://github.com/binfnstats/ukb-IDEARS.*

7. **The authors should have included proper references to the methods and libraries; XGBoost, and SHAP; used in the IDEARs platform.**

*Thanks for raising this and we apologise for this oversight. We have now referenced the original XGBoost paper. The SHAP reference is to* [*https://arxiv.org/abs/1705.07874*](https://arxiv.org/abs/1705.07874) *which we understand to be the correct paper to cite based on this advice* [*https://github.com/slundberg/shap*](https://github.com/slundberg/shap)*, as we use SHAP in a “general sense”. We have added a citation to this paper to the Methods section.*
