# Supplementary material for: Machine learning analysis of the UK Biobank reveals IGF-1 and inflammatory biomarkers predict Parkinson’s disease risk
Source: PLoS One. 2023 May 9;18(5):e0285416. doi: 10.1371/journal.pone.0285416 (PMC10168570; doi:10.1371/journal.pone.0285416)
Supplement: Attachment — Submitted filename: Response To Reviewers_20230402.docx [file pone.0285416.s003.docx]

**Responses to Reviewers**

3. Has the statistical analysis been performed appropriately and rigorously?

Reviewer #1: No

**Response: *Thank you for pointing out the need for us to make a correction given the multiple comparisons, please see below for our full response here.***

6. Review Comments to the Author

Please use the space provided to explain your answers to the questions above. You may also include additional comments for the author, including concerns about dual publication, research ethics, or publication ethics. (Please upload your review as an attachment if it exceeds 20,000 characters)

Reviewer #1: Thank you so much for your responses.

According to the response, the statistical methods used in figures 5, 6, 7, 8, and 9 was unpaired 2-sample t-tests. The results show that the comparison is between 3 groups of PD patients and non-PD and involved several independent variables, which is multiple testing. The unpaired 2-sample t-tests might not be appropriate for the analysis.

**Response: *Thank you for pointing this out and we apologise for failing to apply this originally. Due to your insights here, we have been able to make these changes using the “Benjami-Hochberg correction for multiple comparisons” (reference 18 in revised paper). This has reduced the associated p values and hence the statistical significance of certain variables, but it has not affected the key conclusions of the paper. We feel that thanks to this addition, this section is now more rigorous.***
